# Corneal optical density: Structural basis, measurements, influencing factors, and roles in refractive surgery

**DOI:** 10.3389/fbioe.2023.1144455

**Published:** 2023-04-06

**Authors:** Ye He, Bo-Sheng Ma, Jun-Hao Zeng, Dai-Jin Ma

**Affiliations:** ^1^ Changsha Aier Eye Hospital, Changsha, China; ^2^ Department of Ophthalmology, The Second Xiangya Hospital, Central South University, Changsha, China; ^3^ Xiangya School of Medicine, Central South University, Changsha, China

**Keywords:** corneal optical density, corneal densitometry, pentacam scheimpflug imaging system, FS-LASIK, SMILE

## Abstract

The cornea is the main refractive medium of the human eye, and its clarity is critical to visual acuity. Corneal optical density (COD) is an important index to describe corneal transparency. Intact corneal epithelial and endothelial cells, regular arrangement of collagen fibers in the stroma, and normal substance metabolism are all integral for the cornea to maintain its transparency. In the last two decades, the Pentacam Scheimpflug imaging system has emerged as a breakthrough for the measurement of COD (also called corneal densitometry). It has been found that a wide variety of factors such as age, refractive status, and corneal diseases can affect COD. Different corneal refractive surgery methods also change COD in different corneal regions and layers and affect visual acuity following the surgery. Thus, COD has gradually become a significant indicator to evaluate corneal health, one on which the attention of clinicians has been increasingly focused.

## 1 Introduction

Visual acuity relies on proper transition and focusing of light through the cornea and other tissues before reaching the retina (Brown et al., 2020; Price et al., 2021). Located at the front of the eye, the cornea accounts for two-thirds of the total refraction therein. As such, its optical clarity critically affects the refraction of light and overall optical outcomes of the eye (Patel and Tutchenko, 2019; Mohan et al., 2022). The optical clarity of the cornea depends on its unique structure and biological characteristics, which include intact corneal epithelial and endothelial cells, regular arrangement of collagen fibers in the stroma, and normal substance metabolism (Onochie et al., 2019; Zhang et al., 2019; Mohan et al., 2022).

The measurement of corneal optical clarity began in the 1990s with concerns about the appearance of haze in the cornea following refractive surgery (Andrade et al., 1990). Because early measuring instruments possessed poor sensitivity and image acquisition, the measurement of the optical clarity of the cornea relied mainly on qualitative analysis, which was highly affected by the subjective experience of the clinician (Doughty and Jonuscheit, 2019). The Pentacam Scheimpflug imaging system solves this problem. Through a rotating Scheimpflug camera, the system is capable of assessing corneal optical densitometry (COD) to objectively and quantitatively evaluate the corneal optical clarity (Lou et al., 2022). The range of COD is defined as 0-100. When COD is 0, light can pass through the cornea uninhibited. When COD is 100, light cannot pass through the cornea at all (Takacs et al., 2011).

Recent studies based on Pentacam Scheimpflug imaging system have found that COD can be affected by edema or inflammation, both of which cause structural or biological changes in the cornea in various corneal diseases such as infectious keratitis and keratoconus (Lopes et al., 2014; Orucoglu et al., 2014; Sorcha et al., 2014). Different corneal refractive surgery methods also have distinct effects on COD, and changes in COD after surgery have a certain correlation with visual quality (Lazaridis et al., 2017; Wei et al., 2020; Hou et al., 2022). COD also gradually increases with advancing age in healthy elderly people (Hillenaar et al., 2011). In recent decades, COD has gradually become an important optical index to assess corneal health both in healthy people and in patients with corneal diseases.

## 2 Corneal structure and optical clarity

The cornea is ecial tissue located at the front of the eye with a convex meniscus shape and high transparency. Its equivalent refractive force is about 43 D, accounting for more than two-thirds of the eye’s refraction (Otri et al., 2012; Patel and Tutchenko, 2019). The cornea is an avascular tissue with a complex composition and regular arrangement, and its structure is the key to maintaining its optical clarity (Yu et al., 2022).

### 2.1 Corneal epithelium

The corneal epithelium supports the tear film, protects underlying structures, and maintains the cornea’s transparency (Lavker et al., 2020). The process of oxygen transport and metabolism of the corneal epithelium are vital to corneal clarity. Vicente *et al.* reported that, when the cornea suffered from hypoxia and hypercapnia due to long-term contact lens wearing, the corneal epithelial cells may suffer from acidosis and a subsequent decrease in corneal transparency (Moreno et al., 2022). Molecules such as superoxide dismutase (SOD), glutathione peroxidase (GPX), and catalase (CAT) have been reported to be associated with the normal antioxidant function of corneal epithelial cells (Chen et al., 2009).

### 2.2 Bowman’s layer

Bowman’s layer is an 8-12 μm thick layer formed by non-cellular aggregates that help the cornea maintain its shape (Cankaya et al., 2018). In healthy adults, COD was indicated to be negatively correlated with the thickness of Bowman’s layer (Pekel et al., 2018). Following femtosecond laser-assisted LASIK, areas of focal disruption of the Bowman’s layer were observed to correspond with areas of interface haze, suggesting that decreased corneal transparency is associated with damage to the Bowman’s layer (Vaddavalli Pravin et al., 2012).

### 2.3 Corneal stroma

The corneal stroma is one of the most precisely arranged and transparent tissues in the cornea (Espana and Birk, 2020). The normal arrangement of collagen fibers in the stroma and the stability of stromal cells are critical to corneal clarity. Collagen type I is the primary collagen in the corneal stroma (Song et al., 2021). After corneal refractive surgery, collagen type III will appear (Abdelkader, 2013; Abdelkader, 2016). Compared with collagen type I, collagen type III is thicker in diameter and irregularly arranged (Gandhi and Jain, 2015). The appearance of a large number of collagen type III fibers induces structural changes in the stroma and consequently leads to a decrease in corneal transparency (Gibson et al., 2013).

Following injury of the corneal stroma, the repair process begins with the activation of cytokines to remove damaged cells. Corneal epithelial cells secrete cytokines that induce stromal cells to transform into activated stromal fibroblasts (SFs) (Kowtharapu et al., 2018). SFs have low crystalline contents that lead to a decrease in corneal transparency. In addition, SFs are able to synthesize large-diameter collagen fibers and abnormal extracellular matrix, leading to a higher refractive index and the appearance of corneal haze (Wang et al., 2003). Ha *et al.* have indicated the correlation between corneal haze and COD, and the therapeutic effect of mitomycin C on corneal haze (Ha et al., 2010). Mitomycin C has an antiproliferative effect on corneal SFs that prevent the decrease of corneal transparency (Carlos De Oliveira and Wilson, 2020).

### 2.4 Descemet’s membrane

Descemet’s membrane is a dense, thick, relatively transparent, and acellular basement membrane that separates the posterior stroma from the endothelial layer (de Oliveira and Wilson, 2020). Descemet’s membrane originates from the secretion of endothelial cells at different stages of development and gradually thickens (Eghrari et al., 2015). It plays an important role in the maintenance of corneal transparency as a critical regulatory structure. Together with the endothelium, it is involved in the trafficking of substances, such as transforming growth factor beta (TGFβ) and platelet-derived growth factor (PDGF), that regulate stromal fibrosis and edema and may therefore influence COD (Medeiros et al., 2018).

### 2.5 Corneal endothelium

The corneal endothelium is located on the posterior corneal surface and lacks self-renewal capacity (Kumar et al., 2022). It keeps the corneal stroma in a relatively dehydrated state *via* transporting fluid from the corneal stroma to the aqueous humor, which is critical to corneal transparency (Sie et al., 2020). Tekin *et al.* indicated that COD was significantly correlated with cell density and percentage of hexagonal cells in healthy corneas (Tekin et al., 2017). However, Dorota *et al.* came to a different conclusion in patients with the pseudoexfoliation syndrome (PEX), which is characterized by the excessive production of granular amyloid-like protein fibers in the anterior segment (Tomczyk-Socha et al., 2022). Increased COD was observed in the corneal epithelium in the PEX group, whereas it was not associated with endothelial cell density (Urbaniak et al., 2018). This is possibly caused by the formation of a fibrous layer that is loosely attached to Descemet’s membrane in advanced stages of PEX, and it may increase the COD of the corneal endothelium.

## 3 Measurement methods of the corneal optical density

Due to concerns about corneal haze after refractive surgery, the measurement of corneal optical clarity began in the 1990s (Andrade et al., 1990). In the early stages, the measurement of the optical clarity of the cornea was mainly qualitative analysis using such instruments as the slit-lamp microscope, ultrasound biomicroscope, and optical coherence tomograph (Table 1). Quantitative evaluation of the corneal optical clarity began with the emergence of Pentacam Scheimpflug imaging system (Lou et al., 2022).

**TABLE 1:** Measurement methods of corneal optical clarity.

Methods	Types	Technical principle	Advantages	Disadvantage	Refs
Slit-lamp microscope	Qualitative	Evaluating corneal transparency by haze grading	Simple and practicable in use	Measurement is related to the experience and subjectivity of clinicians, and cannot objectively reflect the degree of corneal clarity	Anumanthan et al. (2017)
Ultrasound biomicroscope (UBM)	Qualitative	B-mode ultrasound	UBM can be used to observe the location, size, and range of corneal haze	Only moderate and above moderate haze can be measured	Kendall et al. (2015)
*In vivo* confocal microscopy	Qualitative	Confocal microscopy	Each sublayer of the cornea can be observed and analyzed by different light intensities	Usually needs to contact the cornea; potentially causes slight shape changes in the cornea, leading to potential measurement errors	McLaren et al. (2016)
Orbscan topography	Qualitative	Placido disc technique combined with the slit scan technique	Can visually display the anterior and posterior corneal surface height, corneal curvature, corneal astigmatism, and corneal thickness	Accuracy is significantly lower than ultrasonic pachymetry	Altan-Yaycioglu et al. (2007)
Optical coherence tomography (OCT)	Qualitative	Based on near-infrared light waves to image microstructures of the cornea	Has a higher resolution and faster speed than that of UBM	Penetrating power is weak and, when corneal opacity is high, is prone to measurement bias	Mori et al. (2009)
Pentacam Scheimpflug imaging system	Quantitative	Based on the Scheimpflug principle, in which the camera and light source speedily rotate and scan the eye	Boasts high resolution, accurate positioning, repeatable measurements, easy operation, non-contact, and quantitative analysis of corneal optical density	Good cooperation of subjects is required to avoid measurement bias	Garzón et al. (2017)

### 3.1 Qualitative measurements of corneal optical clarity

#### 3.1.1 Slit-lamp microscope

The slit-lamp microscope is one of the most widely used instruments in ophthalmic diagnosis. It can be used to observe the corneal haze to evaluate the optical clarity of the cornea (Anumanthan et al., 2017). According to the Fantes scale, the level of haze is graded as: grade 0, completely clear; grade 1, haze can be easily found under the slit-lamp microscope, but it does not affect the observation of iris details; grade 2: haze mildly affects the observation of iris details; grade 3: haze moderately affects the observation of iris and lens; grade 4: the cornea is so cloudy that the iris is totally obscured (Fantes et al., 1990). Although this grading method is simple to use, the grading is often dependent on the experience and subjectivity of clinicians. As such, it cannot objectively quantify the degree of haze. In order to quantify the levels of haze, Hollingsworth *et al.* devised a grading scale to demonstrate alterations in corneal morphology in keratoconus using *in vivo* confocal microscopy (Hollingsworth et al., 2005).

#### 3.1.2 Ultrasound biomicroscopy (UBM)

UBM is able to objectively image the anterior segment of the eye at microscopic resolution (Pavlin et al., 1992). It can accurately determine the size, location, and range of corneal lesions, and it has been widely used in diseases related to changes in corneal clarity, including keratoconus, corneal dystrophy, and corneal scar (Silverman, 2009; Kendall et al., 2015). After photorefractive keratectomy, UBM examination is carried out to document and follow the haze phenomenon (Nagy et al., 1996). However, UBM is only able to assess moderate and above-moderate haze; subtle haze is not observable with UBM (Nagy et al., 1996).

#### 3.1.3 In vivo confocal microscopy

*In vivo* confocal microscopy was first used for the non-invasive assessment of corneal injury and disease at the cellular level by Cavanagh in the 1990s (Cavanagh et al., 1993). *In vivo* confocal microscopy provides multidimensional high-resolution images of the corneal structure at each layer *in vivo*, which reduces the false results caused by specimen processing (Bilgihan et al., 2019). According to the observation of corneal haze, *in vivo* confocal microscopy can determine the structures that contribute to corneal haze at a high spatial resolution (McLaren et al., 2016). In comparison with the Pentacam Scheimpflug camera, *in vivo* confocal microscopy is more suitable for measuring backscatter in the corneas with the highest degree of haze (McLaren et al., 2016). However, the microscope usually needs to contact the cornea; thus the instrument will cause slight shape changes of the cornea in measuring corneal transparency, leading to potential measurement errors. Additionally, while the accuracy of confocal microscopy is high in the central cornea. It is relatively poor in the peripheral cornea (Takacs et al., 2011).

#### 3.1.4 Orbscan topography

Orbscan topographer is a hybrid slit-scanning and Placido disc corneal topographer that can visually display the anterior and posterior corneal surface height, corneal curvature, corneal astigmatism, and complete corneal thickness (Fam et al., 2005; Karimian et al., 2011). It was demonstrated that Orbscan readings were inversely correlated to haze grade (Fakhry et al., 2002; Altan-Yaycioglu et al., 2007). However, its accuracy was significantly lower than ultrasonic pachymetry, suggesting that Orbscan topography alone is insufficient for measuring corneal haze (Altan-Yaycioglu et al., 2007). Moreover, Orbscan topography is a qualitative analysis and thus cannot quantitatively describe the clarity of the cornea.

#### 3.1.5 Optical coherence tomography (OCT)

OCT is a high-resolution optical imaging technique that utilizes near-infrared light waves to image the microstructures of different tissue types (Yang et al., 2022). It was first applied for *in vivo* measurements of the human retinal structure by Swanson et al. (1993). Rahul *et al.* used OCT to assess corneal opacity and found that it could simulate the effect of phototherapeutic keratectomy performed for the removal of corneal opacity (Mori et al., 2009). OCT has a higher resolution and faster speed than that of UBM. However, UBM can better penetrate opaque or cloudy tissues, thereby improving the observation of the ciliary body, posterior iris structures, and anterior chamber in the case of corneal opacity, scarring, and edema (Ursea and Silverman, 2010).

### 3.2 Measurement of the corneal optical density with Pentacam Scheimpflug imaging system

The emergence of the Pentacam Scheimpflug imaging system represents a breakthrough in the evaluation of COD, which is an important quantitative index to evaluate corneal transparency.

#### 3.2.1 Principles of Pentacam Scheimpflug imaging system

**FIGURE 1:**
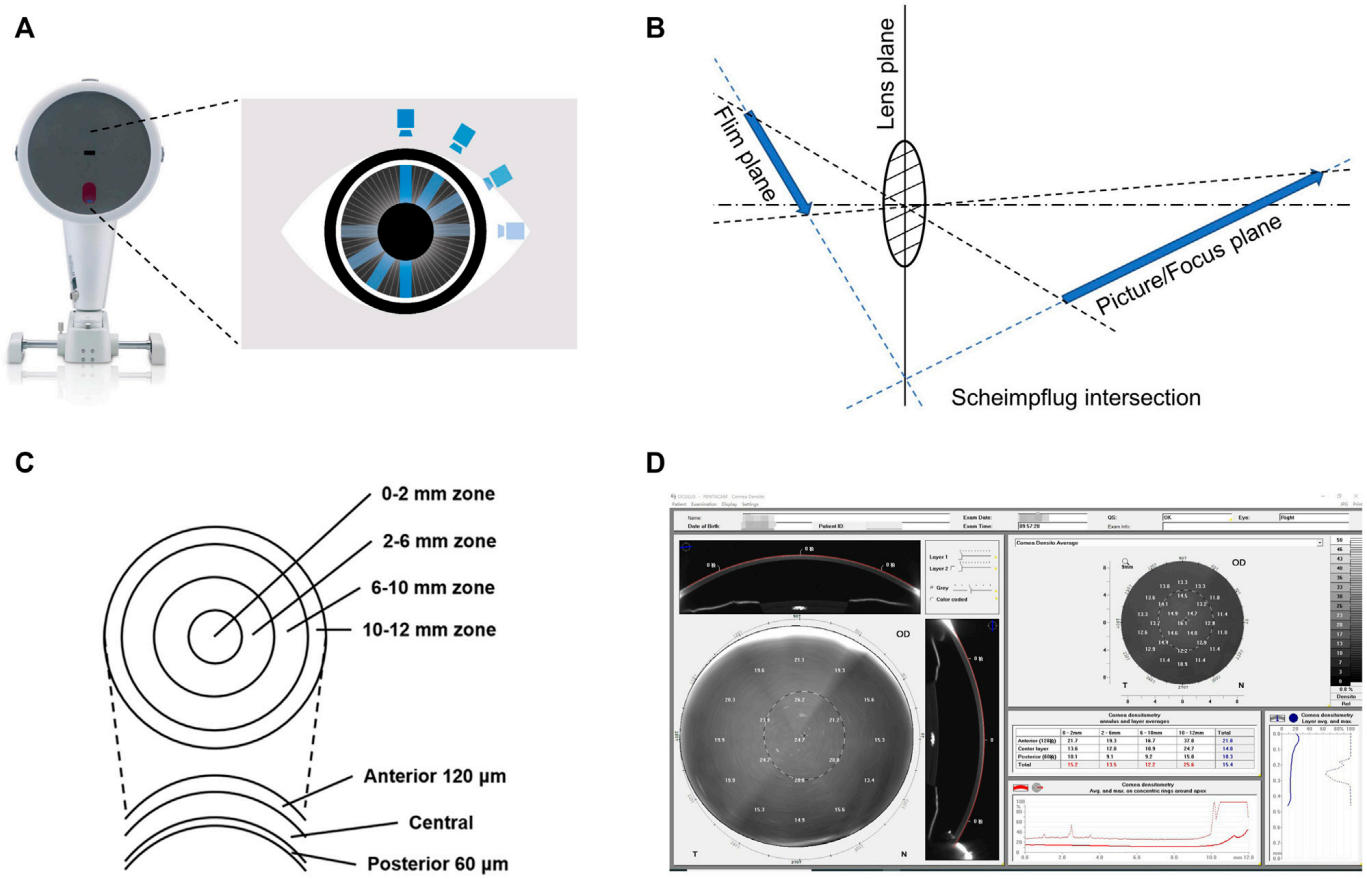
Measurement of corneal optical density by the Pentacam Scheimpflug imaging system. **(A)** Schematic representation of the rotation of the Scheimpflug camera of the Pentacam Scheimpflug imaging system. **(B)** Schematic representation of the Scheimpflug principle. Its main feature is that the plane of the film, the lens, and the picture/focus plane cut each other in a Scheimpflug intersection. Thus, the focused region is increased, and the sharpness of the image is improved. **(C)** Schematic representation of the corneal zones and layers defined by the Pentacam Scheimpflug imaging system. **(D)** Example of the software panel of the corneal optical densitometry in the Pentacam Scheimpflug imaging system. Displayed as a grayscale coded map, the average COD in different corneal zones and layers can be individually assessed and automatically displayed in the table chart.

The system is based on the Scheimpflug principle (Figure 1A, B), in which the rapidly rotating Scheimpflug camera and light source scan the eye, obtaining 50 Scheimpflug images in less than 2 seconds (Efron, 2019). At the same time, using a second pupil camera, eye movements are detected and automatically corrected (Huang et al., 2014; Cavas-Martínez et al., 2016). Pentacam Scheimpflug imaging system measures COD in standardized gray units (GSU) on a scale of 0-100 to quantify corneal transparency at different zones and depths within the cornea (Hsieh et al., 2021).

#### 3.2.2 Method of measuring corneal optical density

During the examination, the patient remains in a standardized dim-light condition for 5-10 min and is subjected to the test in the natural state of the pupil (Pakbin et al., 2022). The patient, who is seated with their chin fixed on a mandibular brace, is asked to stare at a fixed target in the center of the Pentacam blue stripe without blinking or moving their eyes. The examiner selects the measurement mode (usually 25 segments/second) for automatic scanning and acquires data for 2 seconds (Gao et al., 2016). Pentacam Scheimpflug imaging system divides the corneal area into four concentric zones and three layers with different depths (Figure 1C, D) (Li et al., 2021).

## 4 Factors influencing COD in healthy people and patients with corneal diseases

With Pentacam Scheimpflug imaging system, researchers have found that COD varies significantly between healthy people and patients with corneal diseases, as well as between people of different ages and regions (Table 2). Based on emerging evidence, COD has gradually become one significant indicator of corneal health.

**TABLE 2:** Influencing factors of corneal optical density in healthy people and patients with corneal diseases.

Factors	Impact	Descriptions of its impact on corneal optical density	Refs
Ages	Positive	Corneal optical density is significantly correlated with age and corneal thickness; corneal optical density increases with age in the healthy population	Cankaya et al. (2018)
	Non-relative	Corneal optical density is not correlated with age in Chinese patients with myopia	Wei et al. (2020)
Gender	Non-relative	There were no significant differences in corneal optical density between males and females	Sorcha et al. (2014)
	Relative	Females have a higher total corneal optical density than that of men	Garzón et al. (2017)
Regions	Relative	The average value of total corneal optical density in studies from different countries are distinct	Otri et al. (2012), Patel and Tutchenko (2019)
Soft contact lens wear	Positive	Corneal optical density of soft contact lens wearers was significantly higher than that of healthy participants	Ozek et al. (2021)
Orthokeratology lens wear	Positive	Long-term orthokeratology treatment could significantly increase the corneal optical density in young orthokeratology lens wearer	Zhao et al. (2022)
Keratoconus	Positive	Cornea optical density readings of patients with keratoconus were higher than those of the healthy population and correlated with the severity of keratoconus	Shen et al. (2019)
Infectious keratitis	Positive	After pathogenic bacteria invade the cornea, it will lead to a decrease in corneal transparency and a increase in corneal optical density	Orucoglu et al. (2014)
Corneal transplantation	Positive	Corneal optical density in eyes treated by various keratoplasty was significantly higher than that of the normal controls	Koh et al. (2012)
PRK	Positive	The postoperative corneal optical density was significantly higher than that before the surgery	Poyales et al. (2017)
LASIK	Non-relative	After 1 year of LASIK, the corneal optical density did not change significantly	Fares et al. (2012)
	Positive	Corneal optical density of patients with epithelial ingrowth after LASIK is significantly increased	Tian (2018)
FS-LASIK	Positive	Corneal optical density was found only in the peripheral zone (10-to-12 mm annulus) at 1 month after FS-LASIK.	Poyales et al. (2017)
	Negative	A long-term prospective study on FS-LASIK indicated that corneal optical density significantly decreased at 5 years post-surgery compared with the baseline	Wei et al. (2020)
SMILE	Non-relative	At 6-12 months after surgery, the corneal optical density of patients has no significant change from baseline after SMILE.	Wei et al. (2020)
	Negative	Corneal optical density significantly decreased in 3 months and 3 years after SMILE.	Han et al. (2017)

### 4.1 Age and gender

COD was found to increase with age in healthy Caucasians from Belgium (Sorcha et al., 2014). In healthy Spanish participants, change in COD was also positively associated with age in all layers of the cornea (the anterior, central, and posterior layers) (Garzón et al., 2017). However, regarding the concentric zones of the cornea, the change of COD was correlated with age only in the 6-10 mm annulus. In healthy Turkish participants, Ali *et al.* demonstrated a significant positive correlation between total COD and age (Cankaya et al., 2018). With *in vivo* confocal microscopy, Toine *et al.* also found that backscatter in the anterior stroma was significantly increased in healthy participants aged 50 years or older in the Netherlands (Hillenaar et al., 2011). However, Wei *et al.* indicated that COD was not statistically correlated with age in Chinese patients with myopia (Wei et al., 2020). The correlation between COD and age may be caused by the reduction of endothelial cells, which are critical to corneal clarity (Gipson, 2013).

Regarding gender, some studies demonstrate that the CODs of males and females are not significantly different from one another (Sorcha et al., 2014; Cankaya et al., 2018). However, a Spanish study has indicated that women tend to have slightly higher COD (16.60 ± 1.83 GSU) than men (16.22 ± 1.54 GSU) (Garzón et al., 2017). The influence of gender on COD still needs to be further confirmed.

### 4.2 Regional differences

The average values of total COD in studies from different countries are also distinct. A study from the UK showed that the average COD in healthy subjects (64 eyes) was 12.3 ± 2.4 GSU (Otri et al., 2012). A Japanese study (36 eyes) indicated that the average value of total COD in healthy Japanese was 16.4 ± 1.7 GSU (Ozek et al., 2021). A study based on healthy Caucasians from Belgium (794 eyes) indicated that the average COD of the 12 mm-diameter zone was 19.74 ± 3.89 GSU (Sorcha et al., 2014). In Spanish healthy participants (338 eyes), the average COD of the total zones was 16.46 ± 1.85 GSU (Garzón et al., 2017). These distinct results suggest that there may be regional differences in COD. This issue will be addressed by a large-scale international study in the future.

### 4.3 Soft contact lens wear

Soft contact lens wear may cause poor tear film dynamics, inflammatory events, and potential contact lens disease, leading to anatomical and physiological changes in the cornea (Kaido et al., 2020; Mickles et al., 2021; Wang and Jacobs, 2022). A study based on soft contact lens wearers demonstrated that the COD of anterior 0-6 mm annular zones was significantly higher than that of healthy participants; however, the CODs of the 6-12 mm zone in the two groups had no significant differences (Ozek et al., 2021). This change could be due to poor tear function and inflammatory events in the anterior 0-6 mm zone of the cornea, where the soft contact lens interacts with the cornea (Muhafiz et al., 2019).

### 4.4 Orthokeratology lens wear

A number of studies indicated that orthokeratology lens wear could slow the progression of myopia in school-aged children (Hiraoka, 2022). With Pentacam Scheimpflug imaging system, Zhao *et al.* indicated that long-term orthokeratology treatment (about 2 years) could significantly increase the COD of the 0-10 mm diameter area of the cornea in young orthokeratology lens wearers (10.43 ± 2.03 years old) (Zhao et al., 2022). Moreover, COD changes were associated with the fitting mode during the first year, and the COD did not significantly reduce after 1 month of discontinuation (Zhao et al., 2022). This is basically consistent with the effect of orthokeratology lenses, which flatten the central area and steepen the mid-peripheral area in the cornea (Alharbi and Swarbrick, 2003).

### 4.5 Keratoconus

In recent years, COD has gradually become an indicator of corneal health in ophthalmic diagnosis. Lopes *et al.* found that COD was significantly increased in Brazilian patients with keratoconus in comparison with healthy subjects and was positively correlated with the severity of keratoconus (Lopes et al., 2014). A study based on Chinese subjects also reached a similar conclusion (Shen et al., 2019). Additionally, the authors demonstrated that COD values for the anterior layers (0-6 mm), central layers (0-6 mm), posterior layer (2-6 mm), and total layers (0-6 mm) were significantly associated with the stiffness parameter-applanation time 1, which is an important index of corneal rigidity (Shen et al., 2019). Moreover, it was found that the COD of Down syndrome patients with keratoconus showed a significant increase in the middle thickness layer in the 6 mm zone compared to that of Down syndrome patients whose corneas were steeper and thinner than normal (Asgari et al., 2020). Myriam and Ali *et al.* also indicated that, due to the excessive collagen fibers proliferation, the COD of the stromal layer was significantly increased in keratoconus patients after corneal collagen cross-linking (Kim et al., 2016; Böhm et al., 2019; Mahdavi Fard et al., 2019).

### 4.6 Infectious keratitis

In infectious keratitis, corneal ulcers will occur after invasion of viruses, bacteria, and other pathogenic factors into the cornea (Cabrera-Aguas et al., 2022). Faik *et al.* reported that archipelago keratitis led to a significant increase in COD (Orucoglu et al., 2014). Following treatments with antiviral or antibacterial drugs, the infiltrates were able to be reduced within 5 weeks. The initial changes were unable to be observed *via* slit-lamp microscopy, but measurement of the COD allowed the evaluation of therapeutic improvement in corneal clarity, which was decreased from 96.5 to 38.6 GSU (Orucoglu et al., 2014). In bacterial keratitis, COD of the inflamed area was significantly increased. Even after 1 month, when the corneal wound was almost healed, COD was still higher than that of the adjacent normal corneal area (Otri et al., 2012). Therefore, measuring COD with Pentacam Scheimpflug imaging system may serve as a powerful tool to assess the severity of infectious keratitis and the efficacy of drugs therefor.

### 4.7 Corneal transplantation (keratoplasty)

Corneal transplantation is widely used following corneal damage (Català et al., 2022). Complications such as acute rejection and/or corneal infection are the main causes of corneal transplant failure (Armitage et al., 2019). Salvatore *et al.* found that, following Bowman’s layer transplantation, the transparency of the cornea decreased, mostly in the central and Bowman’s layer transplantation posterior layers where the graft had been placed (Luceri et al., 2016). Moreover, a study based on various selective lamellar keratoplasty procedures demonstrated that COD in eyes treated by different keratoplasties was significantly higher than that of normal controls (Koh et al., 2012). These studies suggest that the measurement of COD could provide a basis for clinical diagnosis after corneal transplantation.

## 5 Roles of the corneal optical density after refractive surgery

With the improvement of corneal refractive surgery, most patients can obtain better visual acuity after surgery (Ang et al., 2021). However, due to the degree of laser ablation, corneal haze, poor wound repairing, and other reasons, there is still a small percentage of patients who do not experience the expected outcomes (Jahadi Hosseini et al., 2016; Chang et al., 2022). Corneal transparency and corneal optical density may be affected by damage to the cornea and affect the visual acuity after corneal refractive surgery (Savini et al., 2016; Charpentier et al., 2021).

### 5.1 Photorefractive keratectomy (PRK)

Corneal haze is a major complication following PRK, one that causes decreased visual acuity, refractive regression, and alterations in the quality of vision (Charpentier et al., 2021). Many studies have indicated that COD after PRK is significantly higher than before surgery (Cennamo et al., 2011; Takacs et al., 2011; Poyales et al., 2017). Increased ablation depth during surgery causes more serious corneal damage and a higher COD (Charpentier et al., 2021). At 3 months post-surgery, a decreased level of haze was associated with a reduction in COD (Boulze-Pankert et al., 2016). In order to evaluate the long-term effect of PRK on corneal densitometry, a recent study was carried out based on myopic patient who had photorefractive keratectomy more than 22 years (Montorio et al., 2020). The authors demonstrated that the COD of the anterior layer in the central zone of the cornea was significantly increased in eyes that had been operated on with greater ablation depth in comparison with unoperated eyes (Montorio et al., 2020). However, there was no significant difference in the CODs of eyes operated on with lower ablation depth and unoperated eyes (Montorio et al., 2020).

### 5.2 Laser-assisted in situ keratomileusis (LASIK)

In the past decade, LASIK has been a popular ophthalmologic surgy to correct myopia (Liu et al., 2021). Usama *et al.* reported that LASIK had good visual outcomes and did not significantly alter COD 1 year after LASIK (Fares et al., 2012). This result indicates that LASIK has less effect on corneal transparency than does PRK (Gadde et al., 2020). Some scholars have found that the COD of patients with epithelial ingrowth after LASIK is significantly increased, suggesting that COD could be used as an objective measurement of the level and progression of epithelial ingrowth following LASIK (Adran et al., 2017; Tian, 2018).

### 5.3 Femtosecond LASIK (FS-LASIK)

Following FS-LASIK, Poyales *et al.* reported that significant changes in COD were found only in the peripheral zone (10-to-12 mm annulus) at 1 month after surgery (Poyales et al., 2017). Giacomo *et al.* reported that the COD of myopic patients increased in the 0-10 mm region of the anterior cornea after FS-LASIK, but this situation gradually reversed within 6 months (Savini et al., 2016). However, the changes in COD were still significant in the annular zone ranging from 6 to 10 mm, where the flap edge was located (Savini et al., 2016). The anterior layer and middle layer within the range of 6-10 mm usually mark the edge of the corneal flap. When the basement membrane is damaged, fibrosis will be repaired, potentially resulting in a drop in corneal clarity (Stramer et al., 2003). A long-term prospective study on patients 5 years after FS-LASIK demonstrated that the COD at all corneal zones significantly decreased in comparison with the baseline, indicating that the clarity of the cornea could continue to improve over a long period following FS-LASIK (Wei et al., 2020).

### 5.4 Small incision lenticule extraction (SMILE)

It is not needed to make a corneal flap in SMILE; this permits a smaller incision and thus less damage to the cornea (Jiang et al., 2022). Apostolos *et al.* reported that the total COD at the annular zone of 0-6 mm showed no significant change 3 months after SMILE in comparison with preoperative values (Lazaridis et al., 2017). In addition, the total COD at the annular zone of 0-6 mm showed a weak negative association with lenticular thickness after SMILE (Lazaridis et al., 2017). Another study based on SMILE also indicated a negative correlation between the COD and corneal thickness, showing a significant reduction in postoperative COD 3 months after SMILE (Alio del Barrio et al., 2021). Because the number of keratocytes and collagen fibrils is associated with the corneal optical property, the reduction in COD may be a result of the decrease of overlying stroma that reduces backscatter components (Li et al., 2013; Lazaridis et al., 2017). Furthermore, animal study has shown that SMILE has a low level of postoperative inflammation and keratinocyte reaction after the surgery (Dong et al., 2014).

Some studies have compared the changes of COD after FS-LASIK and SMILE. Wei et al. reported that the change in COD of the anterior and central layers in SMILE was significantly smaller than that in FS-LASIK at 5 years post-surgery (Wei et al., 2020). Hou et al. indicated that, at 12 months post-surgery, patients who underwent SMILE had lower corneal density in all areas than those who underwent FS-LASIK (Hou et al., 2022). However, Shajari et al. came to a different conclusion; they followed up on changes in COD 1 year after SMILE or FS-LASIK and found no significant difference in COD between the two groups (Shajari et al., 2018).

To sum up, COD has been a significant index to evaluate corneal recovery after refractive surgery.

## 6 Conclusion

Corneal clarity plays a key role in visual acuity. To date, COD as measured by Pentacam Scheimpflug imaging system is the most widely used quantitative index for the evaluation of corneal clarity. Age, regional differences, contact lens wear, and corneal diseases can affect COD. COD increases in different regions of the cornea after surface refractive surgery such as PRK and LASIK. The increase of COD after SMILE and FS-LASIK may be closely related to postoperative corneal edema, corneal stromal fiber hyperplasia, and corneal inflammation. In addition, the change of COD in SMILE was smaller than that of FS-LASIK. The different postoperative COD changes between SMILE and FS-LASIK may be related to the distinct level of inflammatory reaction after the two surgeries. In sum, COD is an important indicator to evaluate the severity of corneal diseases and corneal injury and recovery after corneal refractive surgery.
